# Exploring Language Learning as a Potential Tool against Cognitive Impairment in Late-Life Depression: Two Meta-Analyses and Suggestions for Future Research

**DOI:** 10.3390/bs10090132

**Published:** 2020-08-31

**Authors:** Jelle Brouwer, Floor van den Berg, Remco Knooihuizen, Hanneke Loerts, Merel Keijzer

**Affiliations:** 1 Department of Linguistics & English as a Second Language, University of Groningen, 9712 EK Groningen, The Netherlands; f.a.van.den.berg@rug.nl (F.v.d.B.); r.m.knooihuizen@rug.nl (R.K.); m.c.j.keijzer@rug.nl (M.K.); 2 Department of Minorities and Multilingualism, University of Groningen, 9712 EK Groningen, The Netherlands; h.loerts@rug.nl

**Keywords:** late-life depression, language learning, bilingualism, seniors, cognitive function, meta-analysis, systematic review

## Abstract

Late-life depression (LLD) affects about an eighth of community-dwelling seniors. LLD impacts well-being, with loneliness and small social networks being typical. It has also been linked to cognitive dysfunction and an increased risk of developing dementia. Safety and efficacy of pharmacological treatments for LLD have been debated, and cognitive dysfunction often persists even after remission. Various cognitive interventions have been proposed for LLD. Among these, one has received special attention: foreign language learning could serve as a social intervention that simultaneously targets brain structures affected in LLD. Lifelong bilingualism may significantly delay the onset of cognitive impairment symptoms by boosting cognitive reserve. Even late-life foreign language learning without lifelong bilingualism can train cognitive flexibility. It is then counterintuitive that the effects of language learning on LLD have never been examined. In order to create a theoretical basis for further interdisciplinary research, this paper presents a status quo of current work through two meta-analyses investigating cognitive functioning in LLD on the one hand and in senior bilinguals or seniors following a language course on the other hand. While LLD was consistently associated with cognitive dysfunction, inconsistent results were found for bilingualism and language learners. Possible reasons for this and suggestions for future research are subsequently discussed.

## 1. Introduction

The world’s population is aging, both in relative and absolute numbers. Since the early 1900s, the mean life expectancy has increased by more than 30 years in some countries [1]. This increased longevity combined with declining birth-rates and a so-called ‘baby boom’ following World War II means that the proportion of those aged 65 and older will increase significantly in the coming years. This relative aging of the population is accompanied by many societal challenges. After the age of 50, for instance, the cost of healthcare grows exponentially every year [2]. Apart from the increased financial burden, there are also challenges on a personal level: a number of age-specific illnesses reduce cognitive functioning and quality of life. Most pertinently, neurodegenerative illnesses such as Alzheimer’s Disease (AD) are predicted to take over as a primary cause of death in developed countries [3]. The number of AD patients in the United States, for instance, is estimated to grow from 4.7 million in 2010 to 13.8 million in 2050, affecting half the population over 65 [4]. However, while research pertaining to AD is undeniably important, it overshadows a lesser-known threat to healthy aging: late-life depression (LLD).

Falling in the realm of major depression disorder, LLD can be diagnosed on the basis of symptoms stipulated in the *Diagnostic and Statistical Manual* (DSM) [5]. LLD includes both early-onset depressions that persist or recurs after the age of 60 as well as the first diagnosis of depression after age 60 [6]. A diagnosis of depression can follow if an individual currently displays at least one of the two core symptoms: (1) depressed mood or (2) loss of interest/pleasure; and at least four of the following symptoms during at least a two-week period of time: (3) unintended large changes in weight (+/− 5% in a month), (4) insomnia or hypersomnia, (5) psychomotor agitation or retardation, (6) fatigue or loss of energy, (7) feelings of worthlessness or inappropriate guilt, (8) diminished ability to think or concentrate, or indecisiveness, and (9) recurrent thoughts of death or suicidal ideation [5]. Despite limited public awareness, LLD is extremely common: in high-income countries, major depressive disorder was found in 0.9–9.4% of community-dwelling seniors and in 14–42% of those living in care homes; minor depression (the presence of one core symptom and one to three of symptoms 3–9 mentioned above) was found in up to 49% of older adults [7]. Comparable numbers have been attested in low to middle-income countries [8].

Despite a high prevalence of LLD in large-scale epidemiological studies, awareness appears poor, and the illness is often difficult to diagnose. Part of the reason why LLD is so underdiagnosed can be traced back to its symptomatology. Many of the symptoms are somatic in nature, commonly leading older patients and/or their caretakers to mistake these signs for physical illness [9]. A number of studies also suggest that in some LLD patients, especially men, low mood is not necessarily present, further complicating diagnosis [6,10]. Patients suffering from LLD may also feel less inclined to see their general practitioner (GP) for experienced mental health issues, due to stigmas attached to psychiatric disorders [9,10]. Finally, the underrepresentation of LLD may also be associated with healthcare providers. A meta-analysis by Mitchell et al. suggested that GPs often do not take into account the high prevalence of depression in seniors [11]. The study found, for instance, that conversations regarding mental health were quite rare, and often not initiated by the GPs themselves.

The impact of LLD on mental health and cognition is all-encompassing. Not only are mood disorders present in up to 87% of older adults who die by suicide [12], but nonsuicide mortality is also higher in these populations [6]. This can be partially explained through higher incidences of unhealthy behaviors such as smoking and excessive alcohol use among this group [13,14], but also potentially through the lack of social contacts [15]. Additionally, there is a strong and consistent finding that LLD patients suffer from cognitive dysfunction [16,17]. Related to this, LLD significantly increases a person’s risk of developing AD or other types of dementia [18,19]. This consistent association between LLD and cognitive dysfunction has sparked theories that depression is a prodromal form of dementia or that LLD and dementia are underpinned by a similar pathology [20]. An influential review paper by Butters et al. identified a number of possible associations between LLD and AD [21]. One theory they postulated was that depression-related disturbances in glucocorticoids (hormones associated with stress-response, among other processes) lead to hippocampal atrophy, which may increase the risk of developing AD. However, Butters and colleagues proposed that, out of a number of potential pathways, the most common scenario would be that “accumulate[d] AD neuropathology over many years along with co-occurring cerebrovascular disease [i.e., related to blood flow in the brain] damages the frontostriatal circuitry [i.e., the neural pathways connecting the frontal lobe to the striatum], leading to late-life depression” [21]. Another overview paper reached similar conclusions but emphasized that the cause-effect relationship needed to be investigated more thoroughly for a solid understanding [22].

While there is thus some support for LLD as a prodromal manifestation of AD, there are many other (preventable) biopsychosocial causes, associations, and risk factors for LLD that are “complementary and almost always transactional” (i.e., the risk factors interact with each other) [23]. Indeed, external psychological and social variables seem equally important in modulating LLD: seniors with smaller social networks, for example, seem to be more at risk of developing or having LLD [24,25]. Similarly, those who do not have a spouse or confidant develop LLD more often than those who do [15,26,27,28]. Another important factor is the presence of life experiences that build up cognitive reserve. Cognitive reserve can succinctly be described as an individual’s resilience to age-related brain pathology [29]. This reserve can, for example, be built up through higher educational attainment, having (had) a cognitively demanding job, and maintaining cognitively stimulating hobbies later in life [30]. To illustrate, many epidemiological studies investigating LLD reported a higher prevalence of depression in individuals with low educational attainment [24,31,32] and those with less complex occupations during their working life. Likewise, seniors who did not read for leisure were more often depressed, while playing cognitively and socially stimulating card games seemed to protect against LLD [31]. 

Considering the effects of LLD on personal well-being, and the negative prognosis, the need for effective treatment seems both urgent and evident. Current treatment methods can be problematic, however. Antidepressants, such as selective serotonin reuptake inhibitors (SSRIs), are generally deemed safe in younger populations, but seniors have been found to be much more sensitive to negative side-effects [33]. This can largely be attributed to drug-drug interactions between some SSRIs and other medications often prescribed to seniors [34]. Moreover, while successful implementation of pharmacotherapy has been reported in individual papers [35,36], a 2011 meta-analysis found variable evidence for the efficacy of antidepressants [37]. Conversely, a wide range of psychological treatment modalities, such as cognitive behavioral therapy, life review therapy, and problem-solving therapy have been coined for their potential to alleviate late-life depressive symptoms in several meta-analyses [38,39]. However, while these methods are able to improve patients’ mood and well-being, cognitive dysfunction often persists even after remission [40,41,42]. Koenig et al. suggested that this persistent cognitive impairment could indicate a prodromal form of dementia, but acknowledged that patients may have had cognitive impairments before the onset of LLD [41].

Taking into account the evidence synthesized above, maintaining cognitive function or preventing cognitive decline during or after a depressive episode in older age seems difficult, if not impossible. Indeed, current treatment methods do not seem to restore cognitive functioning. In addition, since a pervasive public sentiment exists that aging is synonymous with cognitive decline [43,44], it may follow that not enough neural plasticity remains to improve again. However, lifelong learning initiatives such as the university of the third age [45], underscored by a growing body of research [46,47] have shown that improving cognition in healthy seniors through several training modalities is most definitely possible. In contrast, a number of researchers noted that current LLD treatment methods do little to nothing to combat cognitive dysfunction. In response to this, these researchers approached the treatment of LLD specifically through training cognitive functions. One study [48] did so by employing serious gaming software, targeting cognitive control through multitasking paradigms. Employing a cognitive training modality in depressed seniors was found to result in a significant improvement in mood. This change in mood in the training group was statistically indistinguishable from that in a control group following a recognized treatment modality (namely, problem-solving therapy). Interestingly, however, performance on previously untrained cognitive tests pertaining to working memory and sustained attention improved markedly in the cognitive training group, while no such improvement was found in the conventional therapy group. 

Building on these outcomes, Morimoto et al. developed a computerized cognitive training intervention that used tasks known to specifically train domains associated with treatment-resistant LLD, such as inhibitory control and cognitive flexibility [49]. This intervention was adaptive to the patients’ performance level, such that it was never impossible, but always challenging. In their pilot study, treatment-resistant participants enrolled in a cognitive intervention were compared to depressed controls (not treatment-resistant) receiving a conventional 12-week pharmacological treatment (escitalopram, 20 mg). The researchers reported a statistically significant reduction in depressive symptoms for both groups. Strikingly, however, in the cognitive intervention, this result was obtained after merely four weeks, instead of 12. Additionally, performance on the Stroop task and Trail Making Task Part B (a task in which numbers and letters distributed on a piece of paper need to be connected in ascending order: 1, A, 2, B, 3, C, etc.) increased in the cognitive intervention group, while staying constant in the pharmacological intervention group. While larger-scale studies are needed to consolidate these results, it seems that cognitive training may benefit LLD patients, both in terms of mood and cognition.

While results are promising, criticism regarding these interventions must be acknowledged. One general issue with computerized cognitive training, both for treatment of depression and general improvement in cognition, is that it may “teach to the test” [50,51]: the outcome measures used to assess changes in cognition are too similar to the tasks used in the training. As a result, transfer effects are less meaningful. Another issue is the operationalization of these training programs. Computerized cognitive training often consists of somewhat repetitive tasks, done alone by participants in their own homes. A large-scale study in the Netherlands investigating several exclusively home-based cognitive interventions (e.g., language training, brain-training games) in healthy seniors [52] reported high participant attrition, with over 40% of participants dropping out before the two-month interventions were completed. In response, the researchers halved the duration of the interventions, but this, too, failed to reduce drop-out rates. Olfers et al., therefore, argued that adding a social component (such as chatrooms for participants) could increase adherence. Depressed seniors, in particular, could potentially benefit from a stronger emphasis on interaction and social networks, considering that loneliness is strongly associated with LLD [24,25].

One presently unexplored intervention for LLD that emphasizes this social component, while still training cognitive functions, is language learning. It has long been theorized that people who already know two or more languages (i.e., bilinguals) enjoy a certain cognitive advantage in comparison to monolinguals, as has been found in studies examining, for instance, word learning [53] and conflict processing [54]. Bilinguals essentially juggle two languages at all times: while one language is used, the other needs to be suppressed, with control transferring from the language domain to the general cognitive domain. It does need to be pointed out that the precise mechanisms underlying this advantage have been questioned recently [55] Additionally, depending on the individual bilingual experience, switches between the spoken languages may be frequent. It is theorized that these patterns of constant (de)activation and switching enhance cognitive control (that is, a bilingual experience causes transfer effects to general domains of cognition) [56]. It is also important to acknowledge that a fierce debate on the bilingual advantage has gone on since the inception of the concept. A large meta-analysis from 2018 compiled evidence from 152 published and unpublished studies [57]. While evidence in favor of the bilingual advantage seemed to be present, initially, these effects disappeared after correcting for publication bias. The authors concluded, therefore, that no systematic evidence existed for a bilingual advantage. They also emphasized the need for pre-registration of studies to ensure that negative or null-findings are published, too. Similarly, a large-scale study containing data from 11,000 participants found no conclusive evidence of bilinguals outperforming monolinguals [58]. It is important to point out, however, that individual differences in bilingual experiences were not taken into account in these larger reviews. Indeed, another critical review noted that research on bilingualism often contains methodological flaws that influence results [59], concluding that it is pertinent “to focus more research attention on the individual features of bilingual experience, as this is where the putative cognitive effects stem from”. 

As was mentioned before, bilinguals’ languages are always active, which is theorized to constantly train cognitive control. Indeed, a number of studies have found that a lifetime of bilingualism delays the average onset of Alzheimer’s disease symptoms by multiple years [60,61,62,63]. Additionally, a study comparing bilinguals and monolinguals with AD, who were comparable in terms of cognitive functioning, found that the degree of neuropathology in the bilinguals was more extensive [64]. This suggests that compensatory mechanisms pertaining to cognitive reserve in lifelong bilinguals are much stronger. This does not mean, however, that a positive result can only be found if bilingualism is lifelong. The constant interference that follows from juggling two or more languages makes language learning unique from other types of cognitive training, as any type of activity requiring language will automatically lead to these activation/deactivation patterns, interfering with earlier acquired languages in ways that learning other new skills will not. For instance, one study investigating children looked into the cognate facilitation effect, a well-known phenomenon where co-activation of two orthographically and phonologically similar words (e.g., “dokter” in Dutch and “doctor” in English) leads to faster response times [65]. Interestingly, these researchers not only found this facilitation effect in children who were lifelong bilinguals but also in children who were still learning their second language. Other studies have also suggested that such effects could also arise early in the language learning process of seniors. Indeed, in a 2013 paper, Antoniou et al. argued that learning a foreign language would likely activate those neural networks that decline with aging [66]. This includes networks affected in LLD. Therefore, they proposed that late-life language learning could have neuroprotective effects, effectively training the cognitive functions affected in aging (and LLD). 

Language learning in seniors is a rapidly emerging field. The focus thus far, though, has mainly been on boosting seniors’ cognitive reserve in order to stave off dementia [66]. However, as we have seen, AD and LLD potentially have similar underpinnings [21]. Like Alzheimer’s, LLD is characterized by pathological declines in cognitive function [16]. Similarly, a risk factor for both LLD and AD seems to be reduced cognitive reserve [24,28,31,32] and LLD has even been considered a prodromal expression of AD pathology [20,21]. Successfully ameliorating or reversing cognitive dysfunction in LLD, then, could potentially delay or prevent further cognitive decline. Additionally, learning and using a foreign language is an inherently social activity, especially when done in a classroom setting. A further advantage of classroom language learning is that it potentially tackles the common problems of loneliness and small social networks LLD patients experience [24,25]. Taking away these two risk factors for LLD, then, could potentially improve mood as well (although this is not the focus of the present paper). Published research directly investigating the efficacy of language learning to improve cognition and mood in LLD, is currently non-existent, although work on language learning in seniors is being carried out at the moment of writing, e.g., [67].

## 2. Aims and Objectives

As the field of late-life language learning as a potential therapy for LLD is in its infancy, it is essential to work towards a stronger theoretical and methodological basis for such interventions. This paper contributes to this aim by conducting two separate meta-analyses. First, an in-depth overview of performance on (standardized) neuropsychological tests (including measures of cognitive flexibility, processing speed, working memory, and inhibition) in seniors with LLD will reveal how cognitive domains are (differentially) affected. Subsequently, an overview of studies investigating the performance of bilingual seniors on these same tests will be compiled (both life-long bilinguals and late-life language learners). We acknowledge that it would be more ideal to only include studies on late-life language learners, but the field of third-age language acquisition is still emerging. It was therefore decided to include studies on lifelong bilinguals, too to substantiate claims relating to future research avenues. We expect the overall cognitive functioning of depressed seniors to be less efficient than in healthy controls, but based on evidence for the neuroprotective effect of bilingualism, we hypothesize that bilingual seniors will outperform their monolingual peers on these tasks. It must be stated that the results of these two meta-analyses will not be directly comparable. Indeed, if healthy older bilinguals outperform healthy older monolinguals, it does not necessarily point to the potential efficacy of language learning as an intervention in LLD. Rather, the two analyses should serve to bridge two currently separate fields (i.e., linguistics and geriatric psychiatry) by giving an overview of the status quo in both fields. Evidence from the present meta-analyses, then, will provide the first steps for a theoretical basis for interdisciplinary work targeting language learning as a potential therapy for LLD. 

Additionally, as a secondary objective, critical attention will be paid to methodological choices made in the literature on both late-life depression and late-life language learning, providing a framework for researchers from various disciplines interested in these topics. Based on the results of this review, concrete suggestions for future research will be presented. 

## 3. Methods

### 3.1. Inclusion and Exclusion Criteria

A number of inclusion and exclusion criteria were established to meet the study’s aims. To be included in the literature review on cognitive functioning in LLD, studies had to compare a group of seniors with depressive symptoms or diagnosed depression to healthy controls (e.g., cross-sectional) on the basis of one or more neuropsychological tests from a pre-specified set. This set included a number of widely-used tests tapping into working memory (forward and backward digit span tasks [68]), processing speed (Trail Making Test A [69]), cognitive flexibility (Trail Making Test B [69], Wisconsin Card Sorting Test [70], color-shape switch task [71], letter-number sequencing task [68], digit-symbol substitution task [68]), inhibition (antisaccade task [72]), and episodic memory (Visual Association Task—Extended [73]). Most of the neuropsychological tasks included in the present analyses were selected because they are commonly used and clinically relevant (e.g., the TMT is often used to indicate potential dysfunction in clinical settings [74]). Two tasks were chosen because they were of particular interest to ongoing work (i.e., the color-shape switch task and the VAT-E). All neuropsychological tasks in the test battery. The neuropsychological tasks were also selected because they would be used in ongoing work of the authors. Longitudinal studies on LLD (e.g., randomized controlled trials) were included, but only baseline data were inspected. For the searches looking into bilingualism, all studies comparing older bilinguals to older monolinguals using this same test battery were included, as were intervention studies looking into language learning in seniors.

Other inclusion criteria for eligibility were:

1. Participants were ≥55 years old (Studies on LLD used various cut-off points for a minimum age of inclusion ranging from 45 [75] to 65 [76]. 55 was chosen as a middle ground that primarily identified studies where participants had a mean age of 65 or higher and resulted in more included studies to meet the current investigation’s aims)
2. The study was published in English.

Exclusion criteria were:

3. Participants having a comorbid psychiatric Axis-I disorder (except anxiety, which is highly comorbid with LLD [77]);
4. Participants having a comorbid neurological disorder (e.g, Parkinson’s disease or Alzheimer’s disease) [78,79];
5. Participants reporting a dependency on alcohol, medication, or other substances [80];
6. Participants using benzodiazepines [81] or beta-blockers [82];
7. Qualitative studies;
8. Case studies;
9. Meta-analyses or (systematic) reviews.

In preliminary searches, only studies that fully adhered to the criteria were considered. However, it soon became clear that a large number of potentially relevant studies, especially those in the realm of bilingualism, had not collected (complete) data on medication use and the presence of comorbidities. For this reason, studies that did not collect these data were also included. The cut-off of 55 years, however, was treated as a hard criterion. 

### 3.2. Search Strategies

All searches were done in PubMed and EBSCOhost search engines. Preliminary search strategies aimed to look into broader domains of cognition (e.g., (“late-life depress*” OR “geriatric depress*”) AND (“inhibitory control” OR …). However, due to a large number of results (>8000), it was determined that the searches would be limited to tests used in the test battery of the author’s lab group. The complete search strategy can be seen in Table 1. No limits were set on publication dates. 

### 3.3. Study Selection

After exporting the search results from PubMed and EBSCOhost, title duplicates were removed manually by one reviewer (JB). Then a list of the remaining titles was compiled, such that the reviewer was blind to the authors and year of publication. Subsequently, titles were carefully screened by the same reviewer, such that only those papers which clearly violated one or more exclusion criteria were removed (e.g., titles indicating that the main focus was bipolar depression). As a next step, the same procedure was repeated for the remaining abstracts. A separate list containing only the title, abstract, and notes from the previous screening round was checked for clear violations of exclusion criteria. Full-text papers were then retrieved from the remaining list of articles. This time, the reviewer looked at all inclusion/exclusion criteria, to determine if the paper could be included in the final review selection. If information pertaining to one or more criteria was missing, the corresponding author for the study was contacted. In the absence of a reply, a first reminder was sent after approximately three weeks. A second, and final, reminder was sent two weeks after the first reminder. The reference lists of included studies were also inspected for potentially relevant work. 

### 3.4. Data Collection Process & Data Items

The data necessary to calculate effect size (e.g., mean, standard deviation, and number of participants) were extracted where available. In cases where this was not possible because the necessary data were not reported, the corresponding authors were contacted using the same procedure mentioned in Section 3.3. When these data could not be retrieved, the study was excluded from further analysis. Additional data were extracted to compare the studies’ methods: country where the study was conducted, age of participants, gender distribution, years of education, scores on mini-mental state exams (MMSEs) [83] or the Montreal Cognitive Assessment (MoCA) [84], and the operationalization of depression or bilingualism. 

### 3.5. Risk of Bias in Individual Studies

Risk of bias in individual studies was assessed independently by two authors (JB, FvdB) using the National Heart, Lung and Blood Institute’s quality assessment tool [85]. This tool consists of 14 questions regarding for instance selection bias of participants, validity of the tools used, and blinding of experimenters. This tool was deemed the most suitable, as it enabled the assessment of cross-sectional studies (others were more aimed at RCTs). A highly similar tool also from the NIH was used for the intervention studies [86]. The scoring guide for these tools explicitly states that overall quality should not be determined by summing up the scores per question. Rather, assessors are instructed to consider how much risk of bias each rating of the questions introduces in the individual studies and to determine the study quality based on the presence of any risks (e.g., selection bias, measurement bias, or confounding). Consensus on the ratings was reached after a discussion between the two reviewers. 

### 3.6. Summary Measures & Synthesis of Results

A pervasive issue with systematic reviews is that they employ so-called ‘vote-counting’ methods [87]: if a review, for instance, finds that five out of ten retrieved studies are in line with their hypothesis, they may conclude that evidence is variable. This method is not ideal because it is done purely on the basis of significance; this becomes especially problematic when effect sizes or sample sizes are small, as described in detail by Combs et al. [88]. It is, therefore, recommended that one calculates a pooled average by conducting a meta-analysis [87]. This method reduces the chance of type II errors because it does not simply count *p* values of generally low-powered studies [89]. Additionally, a meta-analysis assigns more weight to studies with more precision (generally those with larger sample sizes) [87]; this also increases the precision compared to vote-counting.

Hedges’ *g* was calculated for each of the neuropsychological tests included in this review, in R (version 4.0.0) [90] with the ‘esc’ package (version 0.5.1.) [91]. A pooled effect size was subsequently calculated for each neuropsychological test with two or more available effect sizes using the ‘meta’ package (version 4.12-0) in R [92]. This same software package was used to create forest plots displaying the overall effect sizes, and funnel plots to show potential publication bias. Since we expected some methodological variation between studies, we opted for a random-effects model [87]. Neuropsychological tests with only one available effect size were reported in a separate table. 

## 4. Results

### 4.1. Study Selection

**Figure 1:**
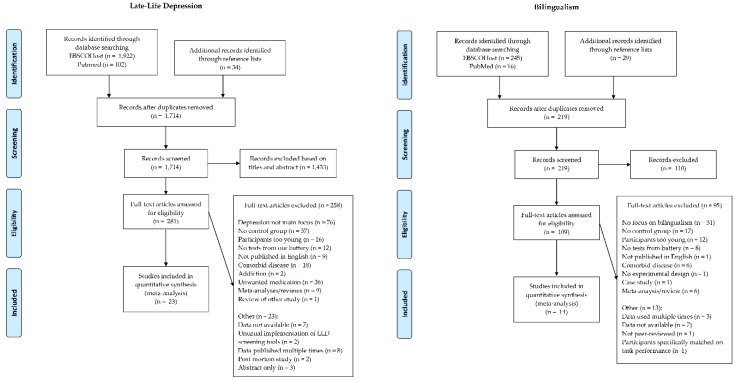
PRISMA flow diagram of the screening process for late-life depression (**left**) and bilingualism (**right**).

The search for papers on cognitive functioning in LLD led to 23 studies that met the inclusion criteria. A total of 14 studies regarding cognition in older bilinguals or older language learners were identified (two of which investigated language learning in seniors). A PRISMA flowchart [93] displaying this process can be seen in Figure 1.

### 4.2. Summary of Study Characteristics Late-Life Depression

An overview of all study characteristics can be found in Table 2, below (for a more detailed version please refer to Table S2 in the supplement). The 23 studies that investigated cognitive function in LLD were published between 1994 and 2018. Two-thirds of these studies (*n* = 14) were published after 2010. The majority of studies were conducted in North America (*n* = 13). Of the other 10 studies, five were conducted in Europe, three in Asia, and two in South America. Six studies contained complete information on all inclusion and exclusion criteria. The 17 studies with missing information did not collect or report information regarding the following: comorbid neurological illness (*n* = 3), comorbid psychiatric illnesses (*n* = 11), substance or medication abuse (*n* = 12), or the presence of medication that led to exclusion in the current design (*n* = 16).

#### 4.2.1. Demographic Information

The 23 retrieved studies contained a total of 4366 depressed participants and 8875 healthy controls. LLD studies had an average of 154.9 depressed participants (*SD* = 359.1) and 385.9 healthy controls (*SD* = 928.9). Groups were not balanced for gender: both LLD and healthy control groups on average comprised more female participants (Depressed: *M* = 67.5%, *SD* = 13.0; Healthy controls: *M* = 64.8%, *SD* = 11.9). Age was explicitly reported in all but one study (which expressed age distribution as the percentage of participants within an age band). Depressed participants were on average 73.9 years old (*SD* = 6.6), while healthy controls had a mean age of 72.3 (*SD* = 5.8). In the 18 studies that reported the total years of education, depressed participants had—on average—one year of education less than the healthy controls (Depressed: *M* = 13.1, *SD* = 4.1; Healthy controls: *M* = 14.1, *SD* = 3.7). Lastly, judging from the 17 studies that included MMSE scores, there was no indication of the presence of global cognitive impairment, and it seemed that depressed and healthy groups were well-matched (Depressed: *M* = 26.9, *SD* = 2.9; Healthy controls: *M* = 26.8, *SD* = 2.5). 

#### 4.2.2. Types of Tasks Used

The following outcome measures were implemented in the included studies: time to complete TMT B (*n* = 13), percentage of perseveration errors on TMT B (*n* = 1), time to complete TMT A (*n* = 13) (One study [94] reported TMT A and B scores in percentiles; these are reported separately), phonemic verbal fluency (*n* = 9), digit span forward (*n* = 9), digit span backward (*n* = 5), (modified) Wisconsin Card Sorting Test categories (*n* = 6) (The modified WCST is shorter and less difficult [95]), (m)WCST perseveration errors (*n* = 4), digit symbol substitution task (*n* = 8), and score on the letter-number sequencing task (*n* = 2). The *n* indicates the number of times a healthy group could be compared to a depressed group. Studies containing multiple comparable groups (e.g., age 65–75 healthy vs depressed and aged >75 healthy vs depressed) were included (and therefore counted) twice.

#### 4.2.3. Operationalization of Late-Life Depression

Nearly two-thirds of the studies (*n* = 14) did not report whether participants in the depressed group had early-onset (before age 60) or late-onset (after age 60) depression. A further five studies reported either only late-onset LLD patients or they provided separate analyses for early and late-onset depression. The remaining four studies reported that they included both early and late-onset patients in their analyses. Blazer states that, while there is some debate on whether there is a difference in etiology between early and late-onset depression, the neuropsychological performance between these two groups does not differ [6]. Therefore, the authors did not differentiate between early and late-onset LLD in their analyses. There was substantial heterogeneity in terms of tools and interviews used to classify depression and depression severity. DSM criteria were used in 14 studies (DSM-III-R, DSM-IV(TR), and DSM-V). Three studies used the International Statistical Classification of Diseases and Related Health Problems (ICD-10), Chinese Classification of Mental Disorders (CCMD-3), and Research diagnostic criteria (RDC), respectively. Most of these studies used additional valid and reliable shorter scales containing between 10 and 30 questions: Hamilton Depression scale (ham-D), the Montgomery Asberg Depression Rating Scale (MADRS), the 30-item or 15-item Geriatric Depression Scale (GDS), the Center for Epidemiological Studies Depression Scale (CES-D), Beck’s Depression Inventory (BDI-II), or the Detection of Depression in the Elderly Scale (DDES). Six of the 23 studies only used one of these shorter scales.

### 4.3. Study Characteristics of Studies on Bilingualism and Aging

An overview of all study characteristics can be found in Table 3, below (for a more detailed version please refer to Table S3 in the supplement). The 14 studies that were identified on bilingualism and aging were published between 1997 and 2020, with the majority (*n* = 11) after 2010. While studies were published in a range of countries (e.g., China, France, Canada), there was a strong bias towards Europe and North-America in participant recruitment. Of the 14 studies, only five studies contained information on all selected inclusion or exclusion criteria. The remaining nine provided no information on comorbid psychiatric illness (*n* = 5), comorbid neurological illnesses (*n* = 5), substance or medication abuse (*n* = 8), or medication status (*n* = 8). Only two studies used language learning interventions in a group of seniors [117,118]. A complete overview of the individual studies can be found below in Table 3. 

#### 4.3.1. Demographic Information

A total of 2147 monolingual and 1225 bilingual participants took part in the 12 identified cross-sectional studies. The two intervention studies had 80 language learners and 117 controls. Studies included on average 102.1 bilingual participants (*SD* = 260.9) and 178.9 monolingual participants (*SD* = 537.2). These averages were heavily influenced by one large cohort study containing 2812 participants (928 bi/multilinguals). Generally speaking, in the cross-sectional studies the bilingual groups comprised the same number of female participants (*M* = 58.1%, *SD* = 24.8) as monolingual groups (*M* = 59.5%, *SD* = 10.2), although it must be mentioned that seven of the selected studies either did not report a gender distribution, or they reported it for all participants instead of for the separate groups. Those studies that did not separately report gender for bilingual and monolingual participants had 58.8% female participants (*SD* = 12.4) on average. In terms of age, monolingual participants in the cross-sectional studies were slightly younger (*M* = 67.8, *SD* = 6.8) than bilinguals (*M* = 70.4, *SD* = 5.5) in the 10 studies that reported age using mean and standard deviation. 

#### 4.3.2. Type of Tasks Used

The 14 studies used the following neuropsychological tasks: Digit span forward (*n* = 6), digit span backward (*n* = 6), digit span forward + backward (*n* = 3) (Because a forward + backward version of the digit span task was present, it was decided to pool all implementations of this task for the meta-analyses.), TMT A (*n* = 4), TMT B (*n* = 4), TMT B–TMT A (*n* = 1) (The effect size for this version was taken together with TMT B measures, as the difference between TMT A and B indexes cognitive flexibility [119]), phonetic verbal fluency (*n* = 4), (m)WCST (*n* = 3), letter-number sequencing (*n* = 2), color-shape switch task (*n* = 1), and the antisaccade task (*n* = 1). None of the studies (including those on LLD) used the VAT-E.

#### 4.3.3. Operationalization of Bilingualism

Substantial variety was attested in terms of how bilingualism was defined. Of the 11 studies comparing monolinguals to bilinguals, one study prompted participants to merely provide the number of languages spoken on a daily basis, without taking into account factors like language proficiency. Only one study used the Common European Framework of Reference for Languages [120] to gauge proficiency. Four studies asked participants for self-reported proficiency measures regarding reading, writing, speaking, and listening. Six studies further supplemented self-rated proficiency with measures regarding, for instance, the frequency and context of use, age or order of acquisition, and language dominance. One of the two studies investigating language learning in seniors required that participants had no previous knowledge of learning another language. The other required that participants had no functional knowledge of the target language (English) but allowed the inclusion of bidialectal participants.

### 4.4. Risk of Bias in Individual Studies

The risk of bias analysis revealed that—out of the 34 identified cross-sectional studies—all but one clearly stated their aims, research questions, and hypotheses. The study populations were in general also adequately described (*n* = 22). Studies that did not accomplish this mostly failed to mention the location from which participants were recruited. Furthermore, whether groups within a study were recruited from similar populations (e.g., from the same time period or location) was unclear in many cases (*n* = 16). Strikingly, only one study reported a justification for their sample size [113]. Many of the studies that did not do this, however, did mention small sample sizes as a limitation. Around half of the studies accounted for different levels of depression or bilingualism in their analyses (*n* = 18). A little over two-thirds of identified studies (*n* = 22) defined their populations consistently (e.g., using the same screening tools). Those that did not screened for LLD on the basis of two versions of the DSM [113], or they used different measures of language proficiency between groups [127]. Having a double-blind study was essentially impossible in most studies due to targeted recruitment of participants with a certain background; therefore, participants were generally aware of their group-status. However, out of all identified studies only one intervention study stated that the experimenters were blinded to group-status of participants [118], and only one cross-sectional study stated that both mood and neuropsychological function were measured by the same clinician [17]. The remaining 35 studies did not mention blinding of experimenters. It must be noted, however, that for many studies investigating bilinguals, blinding experimenters is essentially impossible, since researchers would often know what participants’ first and second languages were. Lastly, around two-thirds of all studies (*n* = 21) controlled for potential confounding variables (e.g., age, gender, years of education), either during the recruitment phase or using subsequent statistical methods. While this might not seem high, only one study had significant group differences at baseline that were not controlled for [110]. The remaining studies did not provide enough information to determine whether confounders were present. For a complete overview of the risk of bias analysis with results for individual studies please refer to Table S3 in the Supplementary Materials.

### 4.5. Cognitive Functioning in LLD and Bilingualism

**Figure 2:**
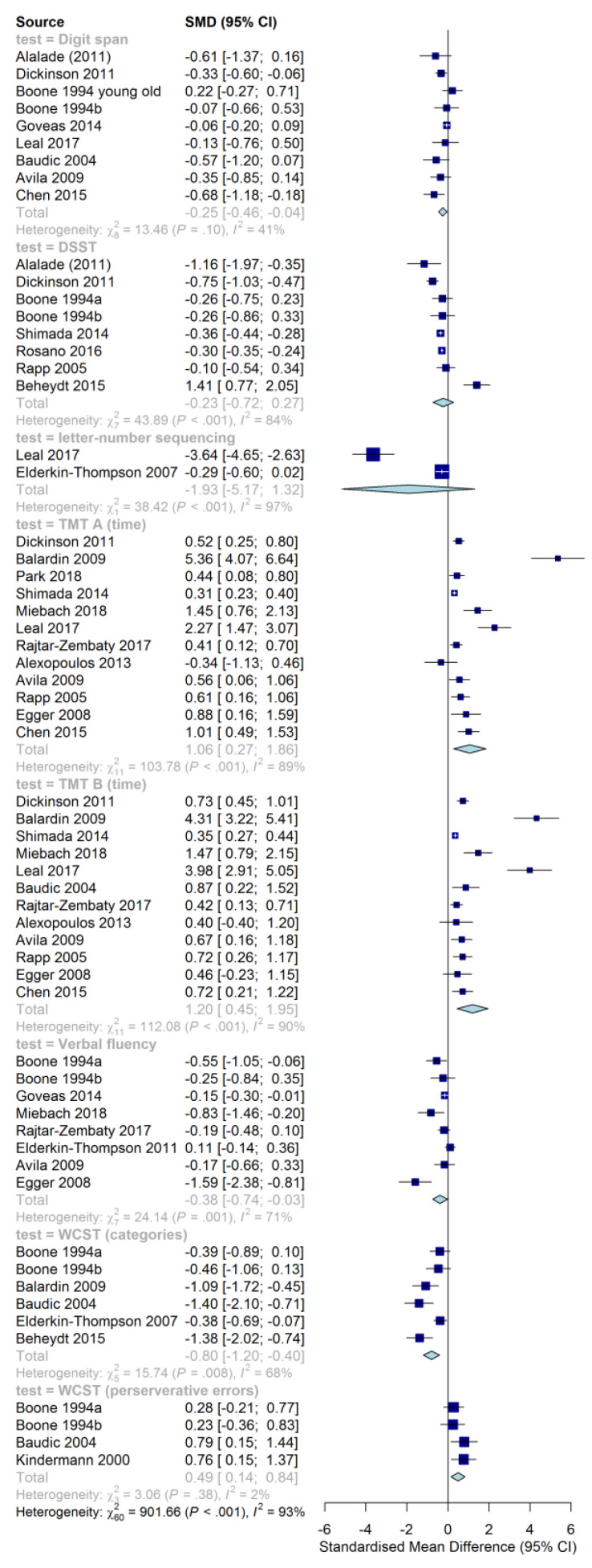
Forest plot for cognitive functioning in late-life depression showing individual and pooled effect sizes. A positive standardized mean difference indicates a higher average score for the depressed group.

The pooled effect sizes for cognitive functioning in LLD are presented in a forest plot in Figure 2. Tasks with insufficient data (i.e, TMT A and B performance measured in percentiles and TMT B perseveration errors) are reported separately in Table 4. Since the two intervention studies looking into late-life language learning used different tests, their results can also be found in Table 4. 

Every square in the forest plot represents a study. The bigger the square, the more it weighs in the pooled effect. The blue diamond represents the pooled effect size (expressed through the standardized mean difference, or SMD). The effect on the left of the null line in Figure 2 indicates a lower average score for the LLD group. A negative effect size generally suggests a disadvantage for the LLD group on that particular task (e.g., fewer categories completed in the (m)WCST implies less cognitive flexibility). However, the opposite is the case for TMT A and B (positive effect size means longer completion times), as well as perseverative errors on the (m)WCST (positive effect size means more errors for the LLD group). 

When looking at the pooled random effect sizes, significant (i.e., with confidence intervals not intersecting zero) small negative effects were found for the digit span task and phonetic verbal fluency. A medium significant positive effect was found for perseveration errors on (m)WCST. Large significant effect sizes were found for TMT A, TMT B, and (m)WCST (categories completed). No significant effects were found for DSST and letter-number sequencing. Overall, judging by the direction of results, the LLD groups seemed to perform worse on neuropsychological tests from our prespecified list.

**Figure 3:**
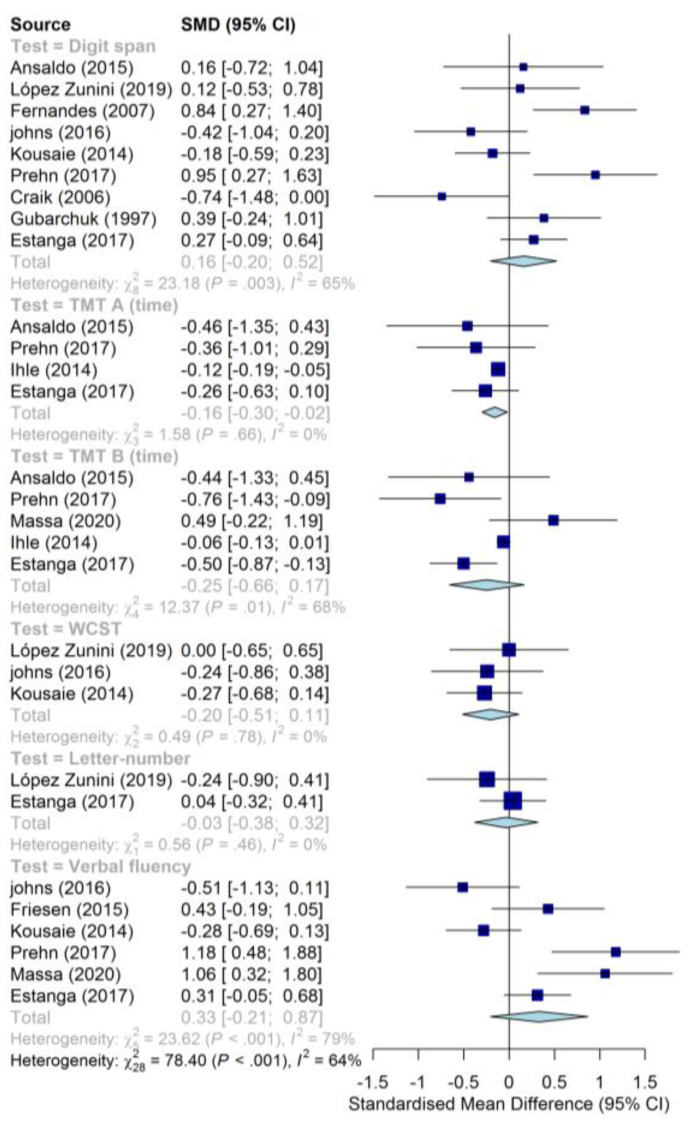
Forest plot for cognitive functioning in older bilinguals showing individual and pooled effect sizes. A positive standardized mean difference indicates a higher average score for the bilingual group.

The (pooled) effect sizes for cognitive functioning in bilinguals can be interpreted the same way (Figure 3): any standardized mean difference (SMD) on the left of the null line indicates a lower average score for the bilingual participants. Again, only for TMT A and TMT B does a negative effect size represent an advantage (faster completion times). The pooled effect sizes for all tasks were small. None of the pooled effect sizes reached significance (i.e., the confidence interval for every test intersected the null line), except for TMT A. In TMT A, a small, significant negative effect was found, indicating that bilinguals completed the task faster than monolinguals. The overview with tasks that were only used once in Table 4 contains three separate groups: studies on late-life language learning, studies on lifelong bilingualism, and studies on LLD. Only performance on the digit-span (backward) was found to significantly improve after a foreign language intervention [118]. The other study found no significant differences following a language course intervention. The studies on lifelong bilingualism reported no significant differences between bilingual and monolingual groups, except for Ansaldo et al.’s study [121], which reported an advantage for bilinguals in terms of accuracy on the TMT A and B with a medium and small effect size, respectively.

### 4.6. Risk of Bias Across Studies

**Figure 4:**
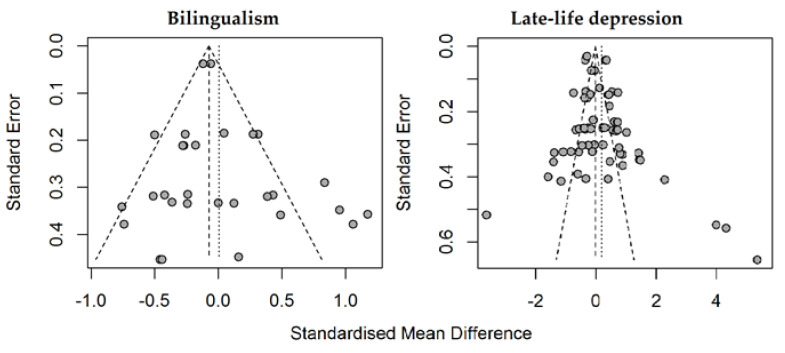
Funnel plots for studies on late-life depression (**left**) and bilingualism (**right**). An asymmetrical distribution suggests publication bias.

Separate funnel plots for studies on LLD and studies on bilingualism were made (Figure 4). The results for LLD, while containing a small number of outliers, were mostly symmetrical, although one study had an abnormally large SMD. Results regarding bilingualism were somewhat asymmetrical, suggesting a publication bias for small studies with larger effects [87].

## 5. Discussion

### 5.1. Cognitive Function in LLD

Our findings indicate that, in line with previous work [16], LLD is consistently associated with cognitive dysfunction. We reported large impairments in processing speed (TMT A), a small to large disadvantage on multiple measures of cognitive flexibility (TMT B, verbal fluency, (m)WCST categories, (m) WCST perseveration errors), and a small deficit in working memory (digit span). For both measures of cognitive flexibility (the DSST and letter-number sequences), mean effect sizes were in line with our expectations, namely that healthy controls outperformed LLD groups. However, the confidence intervals intercepted the null-line, meaning that this effect was not significant. For the DSST, the absence of a significant effect was potentially caused by one study that actually reported a higher number of correct responses for the depressed group. The authors of that study, however, also mentioned that the LLD patients also took more than twice as long to match a digit to a symbol, suggesting an overall processing disadvantage for the depressed group. Only two studies examined the performance of depressed patients and controls on the letter-number sequencing task. One study found a very large negative effect [110], while the other found no significant difference [106]. This led to a large, yet insignificant, pooled effect size. The study by Leal et al. [110] reflects a well-known issue with smaller sample sizes: they potentially overestimate the actual effect size [135]. This overestimation, combined with the small number of studies that used this test, potentially skewed the results. Two studies were not analyzed in the meta-analysis because they reported the TMT differently from the other studies (i.e., completion time expressed as a percentile score [94]; number of perseveration errors on TMT B [116]). Instead, they were analyzed separately. Both studies found depressed participants performed more poorly, as was hypothesized, although only Steffens et al.’s [116] results reached significance. This also provides further support for our hypothesis that LLD patients experience cognitive dysfunction. 

In spite of the generally consistent direction of our findings, most of the results showed substantial heterogeneity (as measured with *I*^2^). Heterogeneity may have been caused by differences between studies in terms of participants’ educational level, present in the studies measuring DSST, for instance. The heterogeneity may also have been caused by underlying group differences not captured in our data. Since the present study dealt with a small number of studies per comparison, heterogeneity was to some extent expected [136]. Additionally, because it was known beforehand that groups would by no means be the same, a random-effects meta-analysis was chosen a priori [87]. This method produces more conservative estimates, which partially counteracts this heterogeneity [137]. 

Despite the small number of studies per comparison, this meta-analysis has provided evidence in favor of our hypothesis, suggesting that generally speaking (non-demented) seniors with depressive complaints perform less well on most measures of cognitive functioning in our selected list of neuropsychological tests. These results, therefore, are also in line with previous research, which reported that seniors with depressive complaints have impaired cognitive functioning, overall [16,40]. 

### 5.2. Cognitive Function in Bilinguals

While the direction of the effects was generally consistent in the analysis on LLD (despite the heterogeneity), the effects of studies investigating cognitive functioning in bilingualism were highly inconsistent. Overall, none of the pooled effects, except performance on TMT A, reached significance, meaning that statistically speaking our meta-analysis did not support our hypothesis. Rather, our results seem to be in line with Lehtonen et al.’s meta-analysis [57]: no statistical support was present for better preserved cognitive functioning in older bilinguals in the identified studies.

Studies specifically looking into language learning in older adults were very sparsely present in our dataset. This can be partially explained by the fact that studies on this topic are scarce, but also because some of the studies on this subject employed neuropsychological tests, not from our predefined list (e.g., [138,139]). Only two studies on late-life language learning could be included [117,118]. These two studies employed no common neuropsychological tests between them, meaning that a pooled average could not be calculated. They will, therefore, be discussed separately. Wong et al. [118] compared a six-month language learning course to a gaming intervention (active control) and a music appreciation intervention (passive control) of the same duration. A small but significant increase was reported exclusively in the language group for working memory, as measured through the backward digit span task. No such increase was reported for the forward digit span. However, the authors did conclude that global cognition, as measured on the basis of the Alzheimer’s Disease Assessment Scale-Cognitive Subscale [140], had improved significantly both in the group learning a foreign language as in those enrolled in the gaming intervention. Ramos and colleagues on the other hand asked participants to complete an eight-month language course [117]. Changes in cognitive flexibility were measured using a color-shape switch task. Reaction times for both switch and non-switch trials decreased considerably between pre-test and post-test in the language training group (by 129 and 157 ms, respectively), while staying relatively constant in a passive control group (69 and 66 ms, respectively). However, the switch cost (the difference in RT between switch and non-switch trials) did not decrease in the language group or the control group. The authors, therefore, concluded that eight months of language training in seniors did not lead to better switching ability, but stated that further research was needed. However, it should be noted that participants in this study could self-enroll for the language course (contrary to Wong et al.’s study, which randomly allocated participants to a group). This may have influenced results. In fact, the language learning group outperformed the control group significantly before the course started, suggesting that the two groups differed at baseline. Lastly, only one type of test was used both at baseline and after completion of the course. The addition of a broader test battery could have provided more nuance.

The findings in this meta-analysis do not support our hypothesis that lifelong bilingualism or late-life language learning enhances cognitive functioning. Only processing speed, as measured through the TMT A, was significantly faster in bilinguals and significantly slower in LLD groups. However, the fact that the results both for the meta-analysis and the individual studies were inconsistent does not mean that the potential for language learning to combat cognitive dysfunction in LLD should be discredited altogether. Our meta-analysis on bilingualism was based on 12 of the 14 identified studies in total, which generally had small sample sizes. Furthermore, the small number of studies (in both the meta-analysis on LLD and on bilingualism) may have decreased the accuracy of the summary statistic; something that will be discussed in more detail in the section on Strengths and limitations. Similarly, rejecting the notion that language learning in late-life may lead to cognitive benefits based on two longitudinal studies would also be an overgeneralization. In looking at the study characteristics in Table 3, we see many variations in terms of how the target demographic was defined. As a result, it becomes difficult to reach conclusions on the basis of the meta-analysis presented above. Instead, it is clear that more research is needed. To ensure that results of future research (both regarding lifelong bilingualism and late-life language learning) are more reliable, the variation present in previous research will be critically evaluated, upon which recommendations for further research will be made.

### 5.3. Issues in Defining Bilingualism

To preface this section, while not strictly relevant to research on late-life language learning, the authors noticed much variation in how bilinguals were described. As much of the work investigating the potential merits of bilingualism in later life compares lifelong monolinguals to lifelong bilinguals, we feel it is important to discuss the term ‘bilingual’. Both the studies on bilingualism and those on LLD used a variety of (diagnostic) criteria and screening tools to differentiate between the two groups they compared. What set studies on LLD apart from the bilingualism studies, however, was their use of validated tools such as the Geriatric Depression Scale and the 17-item Hamilton Depression Rating Scale. These scales were designed to distinguish between healthy and depressed older adults, according to widely accepted DSM and ICD-10 criteria, with high accuracy [141,142]. The studies on language, on the other hand, were much less consistent in defining their monolingual and bilingual participant groups. The term ‘bilingual’ was for the most part operationalized by combinations of proficiency, age of acquisition, and usage patterns. Some studies, for instance, required that bilingual participants had reached a high level of proficiency in the second language before a certain age, which ranged anywhere from 10 to 13 years [123,126,131,132]. What constituted high language proficiency, however, also varied substantially. This was often determined by means of self-reported scales regarding production and reception [129], while only one study used more standardized and objective measures (i.e., based on CEFR guidelines [120]) of language proficiency [134]. Other studies further defined the bilingual groups as those who spoke two languages on a regular basis [123,126,130]. Overall, though, the many ways in which bilingualism was operationalized very likely played a role in the inconsistent results reported above.

These differences between the bilinguals in one study versus those in another are likely caused by the lack of consensus on a ‘case definition’ of bilingualism. In this regard, our findings strongly mirror those reported in a systematic review by Surrain and Luk [143]. They, too, noted that the bilingual experience is a multidimensional construct, consisting of the frequency of use, proficiency, contexts in which languages are used, and sociolinguistic background variables such as the attitudes towards bilingualism in a certain region. In their review of work on bilingualism published between 2000 and 2015, they found that definitions varied considerably. Most studies emphasized proficiency and language use, with less than half using objective measures; this was especially common in studies investigating adult participants. An additional issue was that oftentimes the exposure to languages was presented purely categorically (e.g., whether or not someone spoke multiple languages in the home), instead of on a continuous scale (e.g., percentage of first language usage in the home). As a result, relevant data regarding the situations in which participants used their languages were not present. Surrain and Luk noted that coming to a consensus regarding what makes a bilingual is not realistic. Instead, they argued that our understanding of the cognitive effects of bilingualism would only become stronger when studies included a more complete and transparent description of bilingual experiences. This should include not only proficiency, but also language history, language usage patterns, and sociolinguistic information (e.g., the status of bilingualism in the country where the study took place or personal attitudes towards one’s own bilingualism).

### 5.4. Issues Pertaining to Older Populations Specifically

The inconsistent definition of bilingualism is problematic, but to some extent unavoidable due to the many opinions on what constitutes a bilingual experience and the many (confounding) factors at play in individual bilingual experience. Similarly, in studying older adults, researchers need to take into account certain confounding variables particular to the demographic. One such issue would be the presence of (comorbid) disorders known to affect cognition, such as dementia or mild cognitive impairment (MCI). A majority of the studies on bilingualism (*n* = 10) and those regarding LLD (*n* = 20) that were included in our review employed screening tools for cognitive impairment and dementia such as the MMSE and the MoCa [83,84]. At the same time, nearly a third of the studies on bilingualism (*n* = 4), did not report using such tools, or the included participants self-reported that they were healthy. While they can never be a replacement for a diagnosis by a medical professional, tools like the MMSE are able to indicate if a participant has a cognitive impairment that could act as a confounder. An area where more studies (including those on LLD) were lacking, though, was ensuring that participants were not taking medication that could affect cognitive functioning. Out of the 14 studies on bilingualism, 10 did not have sufficient information available to ensure that participants were not prescribed benzodiazepines or beta-blockers. Additionally, 14 out of 23 studies on LLD did not report on this, either. As mentioned before, this is especially problematic for benzodiazepines [81], which are commonly prescribed to and even abused by seniors [144].

## 6. Directions for Future Research

Results of the present study seem to suggest that, as was hypothesized, cognitive dysfunction in LLD is consistently present. Our meta-analysis also shows that results investigating cognitive functioning in older bilinguals are inconsistent and that no significant evidence was found in our sample that indicated preserved cognitive function in older bilinguals. The authors also noticed that very little research had been done on the effects of late-life language learning on cognition. While the authors of the present study are primarily interested in the role that language learning may have in later life, we realize that cross-sectional study designs are not as resource-intensive as longitudinal designs. For this reason, they are more commonly used to investigate the potential effects of bilingual experiences in seniors. The following section will therefore also give directions for future research comparing lifelong bilinguals to lifelong monolinguals. 

### 6.1. Language Learning Interventions 

Only two studies investigating the effects of late-life language learning on cognition were identified for this review. Given the strong potential of language learning as a cognitive training tool [66], more work is clearly needed. In addition to general methodological improvements mentioned further on, forthcoming longitudinal, research investigating the potential effects of late-life language learning can improve in quality by assigning participants to interventions randomly, like Wong et al. [118]. While this does not completely eliminate the selection bias caused by recruiting participants from the community, it at least negates it somewhat. Additionally, from an applied linguistics perspective, we recommend future intervention studies to tailor their language learning interventions specifically to characteristics, needs, and preferences of seniors [67,145] to maximize the potential benefits of late-life language learning on a motivational, cognitive, and perhaps social (well-being) level. Another important variable that the present analysis did not look into due to a lack of studies on late-life language learners is the appropriate intervention duration. One can imagine that an ideal outcome would follow a multiple year intensive program; indeed, the activities that build up cognitive reserve generally seem to be garnered over a lifetime of repeated exposure [30]. In practice, however, such trials would be incredibly impractical due to participant attrition and budgetary constraints. A 2017 meta-analysis investigated the efficacy of several types of cognitive training in seniors [146]. The interventions these researchers identified consisted of one to 180 sessions and total exposure lasted between 1 and 270 h. Their analysis concluded that 20 or more training sessions administered one or two times per week led to stronger net gains. Their study did not find that intervention duration by itself influenced the effects. Another review investigating primarily the effect of short language learning interventions in mostly student-aged populations found structural changes in brain anatomy (e.g., increased gray matter) after as little as three months of intensive language learning [147]. Improvements in performance on neuropsychological tasks could arise even faster, though. One study reported a sustained improvement in cognitive flexibility (i.e., still present nine months post-intervention) after only one week of intensive language training [139]. In short, the key for optimal results seems to be intervention intensity instead of intervention duration. Further research should focus on an ideal balance between the two. Finally, while this was not the focus of the present study, we feel that researchers in geriatric psychiatry and linguists alike should also study the potential effects of language learning on well-being. While research on this is incredibly sparse at the moment, the few published studies suggest that late-life language learning is an experience that fosters social interactions and feelings of empowerment over one’s aging process [138,148]. 

### 6.2. Lifelong Bilinguals Versus Lifelong Monolinguals

A number of potential reasons may explain the inconsistent findings regarding cognitive functioning in lifelong bilinguals. However, if we start at the root, the inconsistency may be (partly) caused by the operationalization of the term ‘bilingualism’, which ranged from an index of the total number of languages spoken daily (without taking into account dialects or language-proficiency) to more complex measures including frequency of use and context of acquisition. For future research, we recommend using a more transparent operationalization of bilingualism, in line with Surrain and Luk’s recommendations [143], which goes further than, for instance, summing the number of languages a participant reports to speak. This means being more explicit in describing the bilinguals: what languages do they speak, when did they learn them, how do they use their languages, what are their attitudes towards their languages? We believe that using background questionnaires such as the Language Experience and Proficiency Questionnaire (LEAP-Q) [122] and the Language and Social Background Questionnaire LSBQ [128] will help attain this. The information that comes from this, however, is often difficult to use in statistical analyses because resulting the data are quite extensive. An additional, very recently developed, measure that could be useful is language entropy [149]. Entropy essentially takes language background questionnaire data and converts it to an index expressing how balanced participants are in their language use.

### 6.3. General Methodological Improvements

More generally, studies both on bilinguals and LLD were often lacking in the medical information collected and/or reported by the authors. It may be difficult to obtain a participant’s full medical history. However, if it is known whether participants take certain medications, or if they have cognitive impairments as measured through short screening tools such as the MMSE or MoCa, it is at least possible to control for this in analyses. We, therefore, recommend that forthcoming work targetting older bilinguals or seniors learning a language (whether healthy or depressed) records this information, too. On the one hand, this will improve the reliability of new research findings within the field of foreign language acquisition. For instance, it is well-known that long-term benzodiazepine use in seniors leads to considerable cognitive deficits [81]. Including these individuals without controlling for their medication use, then, introduces an unwanted confounder. At the same time, some relatively strong claims regarding bilingualism’s neuroprotective effects are currently being made. The inclusion of these measures will enhance the (clinical) relevance of research on bilingualism for those working in other fields, like gerontology. Controlling for these variables, then, will not only improve overall study quality, but it will also further stimulate understanding and cooperation between disciplines.

Furthermore, based on what was seen in the risk of bias assessment, a number of recommendations for forthcoming cross-sectional studies both on LLD and bilingualism can be made. Firstly, researchers should reduce bias by including power analyses, and by blinding assessors for participants’ group-status as much as possible. If the latter is not possible due to, for instance, the language of a particular task giving away group status, researchers should be more transparent about this. Similarly, bias could be reduced by increasing transparency regarding where and how participant groups were recruited. Again, it may not always be possible to recruit subjects from one clinic. However, a considerable proportion of studies did not provide enough information to determine whether participants were recruited from similar populations. Lastly, while over half of the identified studies reported that the researchers controlled for potential confounding variables (such as age or education level), a large proportion of studies (*n* = 14) did not provide enough information to determine whether this was done. Again, we recommend that researchers explicitly report if participant groups differed on confounding variables and whether they controlled for this in their analyses. 

## 7. Strengths and Limitations

While it has been tentatively proposed that language learning may have a positive effect on cognitive functioning in seniors with depression [66], no study as of yet has bridged the two separate disciplines needed to research this (i.e., linguistics and geriatric psychiatry). A strength of the present research was the use of weighted averages across tests. Vote-counting would have resulted in an outcome that would have seemed more intuitive on the surface, but this method is flawed [87,88,89]. 

In interpreting the results of this study, a number of limitations should be considered, however. Firstly, the search strategies were developed to identify studies using specific validated neuropsychological tests that would likely also be used in ongoing work (including but not limited to our own lab). On the one hand, this was an advantage because it made the studies relatively easy to compare. A disadvantage, on the other hand, was that some relevant studies were excluded because they did not use tests from our predefined list. A study by Bialystok et al. [150], for instance, found that older bilinguals were better at certain types of conflict resolution than age-matched monolinguals. However, because the tests they employed (modified antisaccade tasks) did not match our list, their study had to be excluded. Another shortcoming of the present review was a result of it being conducted during the COVID-19 pandemic. A large majority of the corresponding authors who replied to our queries were more than willing to provide the information requested. However, due to travel restrictions, some were unable to access the data requested. Additionally, since the majority of identified studies did not report or collect data regarding all our inclusion criteria (e.g., medication use), there is a chance that some studies contained undesirable confounders. Regarding medication use, the exclusion of beta-blockers was chosen because reported side-effects include reduced neuropsychological functioning and increased risk for depression [151]. Recent reviews, however, have provided evidence contradicting this notion [152]. In practice, however, the majority of studies excluded for undesirable medication were due to benzodiazepines, which are known to affect cognition significantly [81]. Another limitation was that the selection of publications based on inclusion and exclusion criteria was done by one author (JB). Potential bias caused by this, however, was negated by showing the reviewer (JB) only the title and abstracts. Another major shortcoming is the small number of studies identified. Borenstein et al.’s comprehensive introduction to meta-analyses does, in fact, warn against a lowered accuracy when performing a random-effects meta-analysis on the basis of few studies [87]. However, immediately afterward they also state that “when faced with a series of studies people have an almost irresistible tendency to draw some summary conclusions from them” (i.e., vote-counting). Borenstein et al. therefore suggest that it is preferable to still compute a summary statistic with known shortcomings instead of inviting unknown properties, a procedure followed in the present investigation. The last shortcoming was using studies on lifelong bilinguals in lieu of papers focusing solely on language learning interventions. We argue that bilingualism is a continuum of experiences [153] and that part of the beneficial effect stems from juggling two competing lexicons [154], a process underlying both the language experience of lifelong bilinguals and new learners. This is why we opted to supplement data on late-life language learners with that of older lifelong bilinguals. However, to say that a lifelong bilingual and a late-life language learner are equivalent in terms of cognitive performance is impossible to say based on the limited existing research. 

## 8. Conclusions

A growing body of research is emerging regarding the potential positive effects that bilingualism and language learning may have on old-age disorders [66,155]. These studies have typically focused on building up cognitive reserve across the lifespan in order to stave off clinical symptoms of dementia [60,61,62,63,64]. However, language learning’s potential therapeutic role in late-life depression (LLD), which has been theorized to be a prodromal manifestation of Alzheimer’s [20,21], is underinvestigated. In order to lay out a theoretical basis for future (interdisciplinary) research, this review aimed to provide an overview of the cognitive domains affected in LLD, as well as an overview of cognitive functioning in older bilinguals and seniors learning a language. The results show that despite heterogeneity between studies, LLD was consistently associated with reduced performance on neuropsychological tests. Studies on bilingualism, however, like Lehtonen et al.’s meta-analysis [57] did not show consistent evidence in favor of enhanced cognitive functioning in bilinguals. We believe that this was likely caused by a varying operationalization of bilingualism and a lack of controlling for age-specific confounders. For further research, we suggest taking these potential pitfalls into account. Lastly, it was noticed that the body number of studies on longitudinal language interventions in seniors was very small Aside from a general call to investigate late-life language learning further, the authors recommend that linguists and researchers in gerontology alike be mindful of possibilities reduce their study’s risk of bias through, for instance, randomization of group allocation. 

## Figures and Tables

**Table 1:** Complete search strategy for the present study.

Date	Database	Search String
23 January 2020	PubMed, EBSCOhost	(“late-life depress*” OR “geriatric depress*”) AND (“Trail making t*” OR “TMT” OR “color-shape switch t*” OR “colour-shape switch t*” OR “phonemic verbal fluency” OR “Visual association t*” OR “Vat-E” OR “wisconsin card sorting t*” OR “WCST” OR “modified wisconsin card sorting t*” OR “mwcst” OR “digit-span” OR “digit span” OR “letter number sequencing” OR “letter-number sequencing” OR “symbol coding t*” OR “symbol-coding t*” OR “digit substitution t*” OR “DSST” OR “antisaccade” OR “anti-saccade”)
17 March 2020	PubMed, EBSCOhost	(“senior*” OR “older adul*” OR “third-age” OR “third age” OR “65*” OR “aged” OR “elder*” OR “pension*”) AND (“lifelong bilingualism” OR “bilingualism” OR “language course” OR “language learning” OR “language training” OR “language acquisition” OR “multilingualism” OR “foreign language” OR “language teaching”) AND (“Trail making t*” OR “TMT” OR “color-shape switch t*” OR “colour-shape switch t*” OR “phonemic verbal fluency” OR “Visual association t*” OR “Vat-E” OR “wisconsin card sorting t*” OR “WCST” OR “modified wisconsin card sorting t*” OR “mwcst” OR “digit-span” OR “digit span” OR “letter number sequencing” OR “letter-number sequencing” OR “symbol coding t*” OR “symbol-coding t*” OR “digit substitution t*” OR “DSST” OR “antisaccade” OR “anti-saccade”)

**Table 2:** Study characteristics of individual studies on cognitive function in late-life depression. Demographic information includes where the study took place, participants’ age and gender, educational background, and performance on the mini-mental state examination. Additionally, information is provided on how each study operationalized depression (continued on the next pages).

First Author (year)	Country	Group	N (fem.)	Age (*SD*)	Education (*SD*)	MMSE (*SD*)	Operationalization of LLD	Notes
Alalade (2011) [96]	USA	LLD	11 (10)	64.9 (4.5)	14.4 (3.1)	28.9 (1.7)	DSM-IV criteria + at least some depressive symptoms as measured with MADRS (≥8)	On average early onset 33.5 (18.5)
		HC	18 (11)	71.2 (6.6)	15.8 (2.3)	29.2 (1.2)		
Alexopoulos (2013) [97]	USA	LLD	16 (n/a)	69.0 (5.5)	17.2 (2.3)	29.1 (0.9)	DSM-IV criteria + MADRS ≥ 18	
		HC	10 (n/a)	68.6 (7.0)	16.3 (3.8)	28.5 (1.0)		
Avila (2009) [98]	Brazil	LLD	30 (22)	69.5 (7.5)	11.9 (3.3)	27.4 (2.4)	DSM-IV criteria + (HAM-D + MADRS. No minimum for inclusion)	Only the high education group was used here since low education group had only approx. 3 years of schooling
		HC	33 (26)	68.0 (7.1)	11.9 (3.3)	28.4 (1.7)		
Balardin (2009) [99]	Brazil	LLD	22 (9)	69.4 (1.1)	7.1 (0.9)	27.4 (0.4)	≥5 on the 15 –item Geriatric Depression Scale (GDS)	
		HC	22 (16)	67.8 (1.4)	9.6 (0.6)	27.7 (0.3)		
Baudic (2004) [100]	France	LLD	21 (n/a)	71.8 (8.8)	9.6 (3.3)	28.1 (1.4)	DSM-IV criteria + MADRS > 20	
		HC	19 (n/a)	73.6 (7.7)	13.5 (5.6)	29.7 (0.6)		
Beheydt (2015) [101]	Switzer land	LLD	28 (24)	74.7 (7.6)	n/a	25.5 (3.8)	DSM-IV-TR criteria + 30-item GDS score ≥11	Participants matched on y/of education; MMSE scores in LLD group significantly lower
		HC	20 (15)	72.0 (5.1)	n/a	28.3 (1.4)		
Boone (1994) young-old [102]	USA	LLD	23 (n/a)	64.4 (2.8)	15.6 (2.6)	n/a	DSM-III-R criteria measured with SCID interview + (Ham-D. No minimum for inclusion)	Young-old and old-old presented in the same study
		HC	54 (n/a)	64.4 (2.7))	14.5 (2.4)	n/a		
Boone (1994) old-old [102]	USA	LLD	14 (n/a)	75.5 (5.1)	15.3 (2.8)	n/a	DSM-III-R criteria measured with SCID interview + (Ham-D. No minimum for inclusion)	Gender distribution overall is balanced (53% female)
		HC	51 (n/a)	74.7 (4.1)	14.3 (2.8)	n/a		
Chen (2013) [103]	China	LLD	64 (47)	67.5 (6.0)	8.9 (4.2)	29.9 (0.2)	Chinese Classification of Mental Disorder (CCMD-3) + 30-item GDS score ≥ 11	
		HC	31 (18)	68.2 (8.6)	9.0 (4.0)	29.9 (0.2)		
Dickinson (2011) [104]	USA	LLD	112 (63)	68.7 (6.3)	14.2 (2.5)	27.8 (2.4)	Not mentioned (Ham-D + MADRS used to measure depression severity)	
		HC	101 (73)	70.5 (5.7)	15.4 (1.8)	28.9 (1.3)		
Egger (2008) [105]	Austria	LLD	14 (10)	71.4 (7.5)	9.4 (2.2)	27.2 (1.0)	DSM-IV criteria + 30-item GDS score ≥ 15	
		HC	20 (13)	72.3 (7.7)	10.8 (2.7)	28.6 (0.8)		
Elderkin-Thompson (2007) [106]	USA	LLD	95 (63)	70.0 (7.9)	14.9 (2.7)	29.0 (1.3)	DSM-IV criteria + 17-item Ham-D ≥ 8 (minor LLD) + 17-item Ham-D ≥ 15	Combined minor and major depression
		HC	71 (42)	71.5 (7.6)	14.9 (2.7)	29.4 (1.1)		
Elderkin-Thompson (2011) [107]	USA	LLD	112 (77)	69.0 (7.9)	15.6 (2.7)	29.0 (1.1)	DSM-IV criteria measured with SCID interview + 17-item Ham-D ≥ 15	
		HC	138 (86)	71.0 (7.7)	15.5 (2.7)	29.3 (1.0)		
Goveas (2014) [108]	USA	LLD	204 (204)	≅70	≅65% attended or completed college	Modif. MMSE 96.0 (3.5)	≥5 on the 15 –item Geriatric Depression Scale (GDS)	Women’s health cohort study
		HC	2017 (2017)	≅70	≅ 75% attended or completed college	Modif. MMSE 97.0 (2.7)		
Kindermann (2000) [109]	USA	LLD	25 (16)	73.4 (6.0)	13.3 (2.5)	28.4 (1.8)	Research Diagnostic Criteria + 21-item Ham-D ≥ 17	
		HC	20 (12)	71.2 (6.2)	13.8 (2.5)	29.2 (1.0)		
Leal (2017) [110]	USA	LLD	15 (9)	67.9 (6.9)	13.9 (2.2)	28.1 (0.5)	≥4 on 15-item GDS + ≥ 8 on 21-item Beck’s Depression Inventory II	
		HC	27 (18)	72.2 (7.6)	16.7 (2.2)	28.9 (0.2)		
Miebach (2018) [111]	Germany	LLD	21 (14)	69.4 (8.0)	12.6 (2.8)	27.6 (2.1)	ICD-10 criteria + (15-item GDS. No minimum for inclusion)	
		HC	21 (12)	67.5 (7.2)	15.1 (3.1)	29.0 (1.2)		
Park (2018) [112]	South-Korea	LLD	63 (46)	71.2 (5.1)	7.3 (5.5)	23.6 (4.4)	DSM-IV criteria + (15-item Korean GDS. No minimum for inclusion)	LLD group includes major depression, minor depression, dysthymia, and subsyndromal depression
		HC	59 (29)	70.3 (4.7)	10.2 (5.8)	26.3 (3.5)		
Rajtar-Zembaty (2017) [17]	Poland	LLD	87 (57)	68.1 (6.0)	13.6 (3.3)	n/a	DSM-V criteria + ≥ 7 on 15-item GDS + no depressive episode before age 60	
		HC	100 (61)	66.8 (4.8)	14.6 (3.0)	n/a		
Rapp (2005) [113]	USA	LLD	40 (25)	83.3 (8.6)	10.2 (2.1)	23.1 (4.7)	DSM-III-R or DSM-IV + ≥ 11 on 30-item GDS	Combined early and late-onset LLD
		HC	39 (21)	84.1 (6.8)	9.8 (2.0)	26.0 (3.5)		
Rosano (2016) [114]	USA	LLD	2545 (1633)	74.8 (5.3)	33.3% less than high school	n/a	≥5 on 20-item CES-D (subclinical depression) + ≥11 on CES-D (clinical depression)	Combined subclinical and clinical depression
		HC	2146 (1179)	74.6 (5.4)	23.7% less than high school	n/a		
Shimada (2014) [115]	Japan	LLD	657 (245)	71.4 (4.4)	44.6% < 10 years	26.2 (2.5)	≥6 on 15-item GDS or a diagnosis of depression	Combined depressive complaints group with depressed group
		HC	3695 (1921)	71.5 (5.2)	33.3% < 10 years	26.5 (2.4)		
Smoski (2014) [94]	USA	LLD	30 (n/a)	68.3 (6.3)	n/a	>26	DSM-IV criteria measured with SCID interview + (CES-D. No minimum for inclusion)	
		HC	40 (n/a)	70.8 (7.1)	n/a	>26		
Steffens (2001) [116]	USA	LLD	117 (80)	70.3 (7.2)	14.0 (3.6)	≥24 in 90%	DSM-IV criteria + (HAM-D + MADRS + DDES. No minimum for inclusion)	
		HC	142 (103)	70.2 (6.0)	15.8 (2.6)	≥24 in 90%		

**Table 3:** Study characteristics of individual studies on cognitive function in bilingualism and in language learning interventions. Demographic information includes where the study took place, participants’ age and gender, educational background, and performance on Montreal cognitive assessment or the mini-mental state examination (continued on next page).

First Author (Year)	Country	Group	N (fem.)	Age (*SD*)	Education (*SD*)	MOCA/MMSE (*SD*)	Bil. Lang.	Operationali-zation of Bilingualism	Other Language Tests	Notes
Ansaldo (2015) [121]	Canada	Bi	10 (n/a)	74.2 (7.4)	17.2 (3.1)	MOCA 27.2 (1.6)	French-English	LEAP-Q [122]; ≥30% L2 usage	Bilingual aphasia test part C	
		Mono	10 (n/a)	74.5 (7.1)	16.1 (3.28)	MOCA 27.7 (1.1)				
Craik (2006) [123]	Canada	Bi	15 (n/a)	68.8 (6.1)	15.3 (3.7)	n/a	Varied	Speaking two languages daily from childhood (≤10 years)	Context of acquisition; frequency of L2 usage	
		Mono	15 (n/a)	70.3 (4.3)	16.1 (3.5)	n/a				
Estanga (2017) [124]	Spain	Bi	88 (46)	60.5 (4.3)	14 (4)	MMSE 28.7 (1.23)	Basque-Spanish	Speaking two languages regularly and fluently	Semi-structured interview; Bilingual Language Profile questionnaire [125]	Authors sent a subset of dataset that adhered to inclusion criteria
		Mono	43 (25)	60.8 (4.4)	12 (2)	MMSE 28.4 (1.31)				
Fernandes (2007) [126]	Canada	Bi	26 (n/a)	69.7 (0.8)	16.3 (0.5)	n/a	Varied	Speaking two languages regularly from adolescence (≤12 years)	Self-rating reading, speaking, listening, writing (1–10); AoA; frequency of L2 usage; language preference	
		Mono	16 (n/a)	74.1 (7.5)	15.5 (0.5)	n/a				
Friesen (2015) [127]	Canada	Bi	21 (n/a)	71.1 (3.8)	n/a	MMSE >26	Varied	Speaking two languages fluently on a daily basis	Language and Social Background Questionnaire [128]; self-rated prof.; frequency of language usage; context of language use	
		Mono	20 (n/a)	70.9 (2.6)	n/a	MMSE >26				
Gubar-chuk (1997) [129]	USA + Russia	Bi	20 (11)	70.0 (6.6)	13.7 (4)	n/a	Russian-English	n/a	Self-rating of overall English proficiency (1–4)	Data from two experiments in the same study combined
		Mono	20 (10)	68.2 (7.5)	12.0 (0)	n/a				
Ihle (2016) [130]	Switzer-land	Bi	928 (n/a)	≥65	n/a	n/a	Varied	Speaking ≥ 2 languages regularly (regardless of proficiency)		Dialects did not count as languages
		Mono	1884 (n/a)	≥65	n/a	n/a				
Johns (2016) [131]	Canada	Bi	28 (7)	70.6 (5.7)	16.1 (1.27)	MOCA 27.7 (1.3)	French-English	Reaching high proficiency in L2 in early adolescence (≤13 years)	Self-rating reading, speaking, listening, writing (1–5)	
		Mono	16 (10)	74.1 (7.5)	15.1 (3.3)	MOCA 27.1 (1.9)				
Kousaie (2014) [132]	Canada	Bi	36 (17)	70.7 (5.9)	16.1 (2.9)	MOCA 27.5 (1.6)	French-English & English-French	Reaching high proficiency in L2 in early adolescence (≤ 13 years)	Self-rating reading, speaking, listening, writing (1–5)	Combined two monolingual groups for our analysis
		Mono	61 (23)	72.4 (6.5)	15.7 (2.7)	MOCA 27.5 (1.5)				
López Zunini (2019) [133]	Canada	Bi	18 (10)	71.4 (4.0)	16 (2.6)	27.6 (1.6)	French-English	Highly proficient in both languages; no functional knowledge of other languages	Self-rating reading, speaking, listening, writing (1–5)	
		Mono	18 (11)	71.7 (3.5)	15.6 (2.7)	27.8 (1.3)				
Massa (2020) [119]	France	Bi	16 (n/a)	72.3 (5.0)	16.0 (2.7)	MMSE 29.5 (0.8)	French-Italian/Italian-French	Bilinguals were all dominant in French	Sociolinguistic questionnaire regarding frequency/context of usage	
		Mono	16 (n/a)	71.1 (5.9)	15.1 (2.4)	MMSE 28.9 (1.6)				
Prehn (2017) [134]	Germany	Bi	19 (n/a)	>55	Most ≥ Undergraduate	MMSE 29.7 (0.5)	English-German & Russian-German	≥ B2 on CEFR measured with Goethe Institute Placement Test	German vocabulary and grammar test using cloze test	
		Mono	18 (n/a)	>55	Most ≥ Undergraduate	MMSE 29.3 (0.9)				
**First author**	**Country**	**Group**	**N (fem.)**	**Age (*SD*)**	**Education**	**Global cogn.**	**Language learnt?**	**Intervention**		
Ramos (2017) [117]	Spain	Lang	26 (12)	67.4	92% ≥ secondary education	MMSE 28.4	Basque	8-month language course by an education center for adults		
		Ctrl	17 (9)	69.2	71% ≥ secondary education	MMSE 28.5				
Wong (2019) [118]	China	Lang.	53 (43)	70.8 (6.0)	Most < high school graduate	ADAS-Cog 8.3 (4.6)	English	5 h per week 2 to 3 days a week for 6 months in senior center (130 h max)		
		Game	51 (44)	71.1 (6.5)	Most < high school graduate	ADAS-Cog 9.1 (5.4)				
		Music	49 (43)	71.1 (6.1)	Most < high school graduate	ADAS-Cog 9.1 (5.6)				

**Table 4:** Effect sizes of neuropsychological tasks that were used only once.

Author	Group	Test	Hedges’ *g*	Conclusion
Wong (2019) [118]	Language learning	Digit-span (forward)	0.09 (very small) language vs. gaming 0.04 (very small) language vs. music	No significant differences between language/music/gaming groups after completing the intervention
Wong (2019) [118]	Language learning	Digit-span (backward)	−0.26 Language vs. games (small) −0.58 language vs. music (medium)	Significant gains in digit-span performance in language learning intervention, but not in the music or gaming interventions
Ramos (2017) [117]	Language-learning	Color-shape switch (RTs to switch trials)	0.1 (small)	Decrease in RTs on switch trials was slightly larger in language learning group than in controls, but this was not significant
Massa (2020) [119]	Lifelong biling.	Antisaccade (% correct)	−0.50 (medium)	Bilinguals made slightly more mistakes than monolinguals, but this was not significant
Massa (2020) [119]	Lifelong biling.	Antisaccade (congruent –incongruent)	0.18 (small)	Bilinguals had slightly shorter response times than monolinguals, but this was not significant
Ansaldo (2015) [121]	Lifelong biling.	TMT A errors	−0.73 (medium)	Bilinguals made fewer errors than monolinguals
Ansaldo (2015) [121]	Lifelong biling.	TMT B errors	−0.33 (small)	Bilinguals made fewer errors than monolinguals
Smoski (2014) [94]	LLD	TMT A (percentile)	−0.09 (small)	LLD took slightly longer to complete TMT A than HC, but this was not significant
Smoski (2014) [94]	LLD	TMT B (percentile)	−0.47 (medium)	LLD took longer to complete TMT B than HC, but this was not significant
Steffens (2001) [116]	LLD	TMT B (% perseveration errors)	0.27 (small)	LLD group made significantly more perseveration errors than HC

## Supplementary Materials

Supplementary materials can be found in the open science framework (https://osf.io/x3wqz/?view_only=627012de77b44e729cc3c04dcaab534f). They consist of an overview of the selection procedure for both the search on bilingualism (S1) and LLD (S2), as well as a complete overview of the risk of bias assessment (S3).
